# Remote Microwave and Field-Effect Sensing Techniques for Monitoring Hydrogel Sensor Response

**DOI:** 10.3390/mi9100526

**Published:** 2018-10-17

**Authors:** Olutosin Charles Fawole, Subhashish Dolai, Hsuan-Yu Leu, Jules Magda, Massood Tabib-Azar

**Affiliations:** 1 Livanova Inc., 100 Cyberonics Blvd, Houston, TX 77058, USA; olutosin.fawole@gmail.com; 2 Electrical and Computer Engineering Department, University of Utah, Salt Lake City, UT 84112, USA; subhashish.dolai@utah.edu; 3 Chemical Engineering Department, University of Utah, Salt Lake City, UT 84112, USA; u1011614@utah.edu (H.-Y.L.); magda@chemeng.utah.edu (J.M.); 4 Bio Engineering Department, University of Utah, Salt Lake City, UT 84112, USA

**Keywords:** smart hydrogels, bio-sensors, chemo-sensor, electrochemical sensors, transduction techniques, near-field microwave, microwave resonator, microwave remote sensing, potentiometric sensor, gold nanoparticles, metal oxide field-effect transistor, chemo-FET, bio-FET

## Abstract

This paper presents two novel techniques for monitoring the response of smart hydrogels composed of synthetic organic materials that can be engineered to respond (swell or shrink, change conductivity and optical properties) to specific chemicals, biomolecules or external stimuli. The first technique uses microwaves both in contact and remote monitoring of the hydrogel as it responds to chemicals. This method is of great interest because it can be used to non-invasively monitor the response of subcutaneously implanted hydrogels to blood chemicals such as oxygen and glucose. The second technique uses a metal-oxide-hydrogel field-effect transistor (MOHFET) and its associated current-voltage characteristics to monitor the hydrogel’s response to different chemicals. MOHFET can be easily integrated with on-board telemetry electronics for applications in implantable biosensors or it can be used as a transistor in an oscillator circuit where the oscillation frequency of the circuit depends on the analyte concentration.

## 1. Introduction

**Figure 1:**
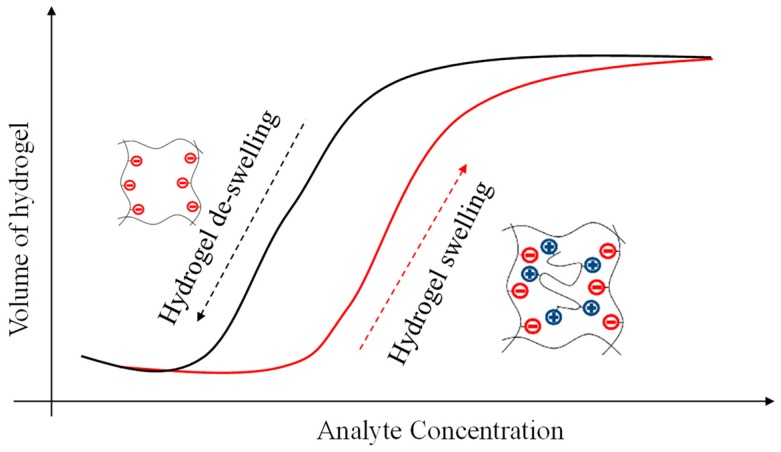
Schematic of a hydrogel functionalized with pendant negatively-charged molecules that attract or release positively-charged target analytes, resulting in physical, electrical, and chemical changes in the hydrogel.

Chemical, biological, and gas sensors usually rely on sensing materials that change their electromagnetic and physical properties in response to molecules and chemicals of interest (analytes). These changes are then measured with electric, magnetic, optical, thermal, or mechanical techniques to identify and quantify the analytes. Hydrogels and aerogels are nearly ideal sensing materials because they contain large voids and open regions (cages) in their structures. These voids allow liquid and gas molecules to diffuse in/out of the hydrogel interior thereby resulting in physical, chemical, electromagnetic, and mechanical changes in the hydrogel. Hydrogels’ cage-like structures comprise cross-linked polymer networks that can be manipulated to interact with molecules with different shapes, ionic content, and pH [1,2]. Moreover, at the microscopic and nanoscopic level, the hydrogel structure can be modified with functional groups for sensitive and selective interactions with specific molecules. Figure 1 schematically shows a hydrogel functionalized with pendant negatively charged molecules that attract a target positively-charged analyte. The combination of these two oppositely-charged molecules results in dimensional, electrochemical, and physical changes in the hydrogel.

Interesting applications of hydrogels include the sensing of important biological analytes such as glucose. Hydrogels respond to stimuli by gradually absorbing/releasing water as shown in Figure 1. The absorption of water by these hydrogels results in an increase in hydrogel volume (swelling) while the release of water results in the decrease (de-swelling) of hydrogel volume. Hydrogel swelling and de-swelling also result in electrical, chemical, and optical changes in the hydrogel.

Various transduction techniques, such as optical, electrical, and mechanical techniques, have been developed in the past to monitor hydrogel response to chemicals and biomolecules [3,4,5,6,7,8,9,10,11,12]. The optical transduction technique measure changes in optical and dimensional properties of the hydrogels with optical methods. This technique can detect small dimensional changes (at a nanometer scale) of hydrogel, as well as small refractive index changes of the hydrogel when the hydrogel responds to chemicals and stimuli. However, the major drawback of the optical technique is the bulkiness of the readout instrumentation needed to capture the optical response of the hydrogel. The electrical technique for measuring the hydrogel’s response to chemicals and stimuli relies on measuring hydrogel conductance [11], resistance or impedance [12]. This electrical technique has the advantage of high sensitivity and it can be used with compact read-out instrumentation. However, the technique requires the hydrogel to be physically connected to an electronic circuit in applications where it is to be applied. Mechanical techniques use strain gauges to measure changes in the hydrogel’s Young’s modulus or volume/density. The main advantage of the mechanical technique is its simplicity. However, a major disadvantage of this technique is that the output signal from the mechanical transducers is prone to drift.

Microwave techniques have been used to measure and monitor changes in the electromagnetic properties of materials [13,14,15,16,17,18,19,20,21,22,23]. Microwave measurements can be very sensitive and can be used remotely through many different media to non-invasively monitor the sensing material’s response to its environment. The intervening media can be air, dielectric layers, and biological tissues (skin, fat, etc.) without the probe making physical contact with the hydrogel or the enclosing media. Microwave wavelengths can vary over a wide range from 100 cm (300 MHz) down to 30 µm (10 THz) providing a powerful monitoring technique with a wide range of spatial resolutions and penetration depth. We are in the process of performing terahertz reflectometry [24,25] and high-spatial resolution measurements of hydrogels, and these results will be reported in the near future.

This paper demonstrates for the first time the remote microwave monitoring of hydrogels in real time. In order to verify that the hydrogel that was synthesized for this study is responsive to analytes and to check the feasibility and accuracy of the microwave technique, we measured the impedance of the hydrogel sample in contact mode. Impedance spectroscopy is a well-known method for the electrical characterization of materials and is extensively used in hydrogels [8,10,12]. Subsequently, we developed “in contact” and remote microwave methods for hydrogel monitoring.

We also developed a potentiometric (measured the potential across the hydrogel with current ~0) technique to monitor the changes in ionic content of the hydrogel. The potentiometric approach was used in the past to construct hydrogel-based batteries and piezoelectric transducers [26,27] but was not used as a transduction technique for hydrogel sensors. Here, the electric potential across the hydrogel was used to detect the ionic concentration gradient across the hydrogel due to an analyte. This phenomenon was then exploited to design a novel metal-oxide-hydrogel field-effect transistor (MOHFET) whose channel material is a smart hydrogel. The MOHFET [28] design was based on an open-face Field-Effect Transistor (FET) structure reported before [28,29,30,31]. Here, drop-casting was used for depositing the hydrogel channel. The MOHFET characteristics changed as a function of the hydrogel’s ionic content. Furthermore, a MOHFET with gold nanoparticles, (AuNP)-embedded, was developed [32]. This gold-doped hydrogel channel increases the conductivity of the MOHFET channel compared to the undoped MOHFET channel. MOHFETs are of great interest because they can be used to detect the microscopic biomolecules in media with high ionic concentrations without the limits imposed by the double electrical layer shielding the effect of the ionic medium (Debye-length free sensing) [33]. The threshold voltage of the MOHFET reported in this paper varied reproducibly with the concentration of the analyte that was measured.

## 2. Materials and Methods

### 2.1. Hydrogel Synthesis

#### 2.1.1. Reagent Materials for Hydrogel Synthesis

For the synthesis [2,32] of the hydrogel, acrylamide (AAM) was obtained from Fluka Analytical. *N*-[3 (dimethylamino)propyl]acrylamide (DMA) was purchased from Polysciences Inc. (Warrington, PA, USA), and 3-acrylamidophenylboronic acid (3-APB) was purchased from Frontier Scientific (Logan, UT, USA). *N*,*N*-methylenebisacrylamide (BIS), 4-(2-Hydroxyethyl)-1-piperazineethanesulfonic acid (HEPES), dimethyl sulfoxide (DMSO), ammonium persulfate (APS), and *N*,*N*,*N*′,*N*′-tetramethylethylenediamine (TEMED) were purchased from Sigma-Aldrich (St. Louis, MO, USA). For hydrogel testing in a medium with high ionic concentrations, 1X-PBS was prepared from Dulbecco’s phosphate buffered saline powder with pH and ionic strength adjusted to 7.4 and 155 mM, respectively. For hydrogel testing of an analyte with low ionic concentration, deionized (DI) water was used for testing.

#### 2.1.2. Synthesis of Stimulus-Responsive Hydrogel

The hydrogel was a polyampholytic copolymer with a nominal monomer composition of 80 mole% AAM, 10 mole% DMA, 8 mole% 3-APB, and 2 mole% BIS. The resulting hydrogel had a composition optimized for measuring blood glucose levels in diabetic patients and it also had a large sensitivity to ionic concentrations [34]. This hydrogel was synthesized by the free-radical crosslinking copolymerization method using APS/TEMED as the free radical initiator [35]. Pre-gel solutions were made by dissolving 19.1 mg of 3-APB powder in 85 μL DMSO in a 1.5 mL centrifuge tube, then adding appropriate amounts of AAM and BIS stock solutions followed by DMA. Monomer mixtures were diluted with additional HEPES to obtain 13 wt% (monomer/solvent) pre-gel solutions. TEMED was added to pre-gel mixtures right before polymerization. Pre-gel mixtures were mixed well using a vortex mixer and purged with Nitrogen gas for 5–10 min. After that, APS was added in an amount equal to 0.2% of the total molar concentration of monomers. The mixtures were mixed for 10 s and then quickly injected into the mold. Molds for hydrogels were constructed from two 8 cm × 8 cm hydrophobic glass slides separated by a 400 μm Teflon spacer. Hydrogel mixtures were left in the mold for 12 h at room temperature. After polymerization, the hydrogels were removed from molds and washed with deionized (DI) water in order to remove excess monomers. The hydrogels were cut into cylindrical disks of diameter 5 mm. The thickness of each hydrogel disc was 400 μm. Next, the hydrogels were alternatively washed in 1X-PBS solutions and 0.5X-PBS solutions (three cycles), and then stored in 1X-PBS at ambient conditions in a dark place until experimental testing.

**Figure 2:**
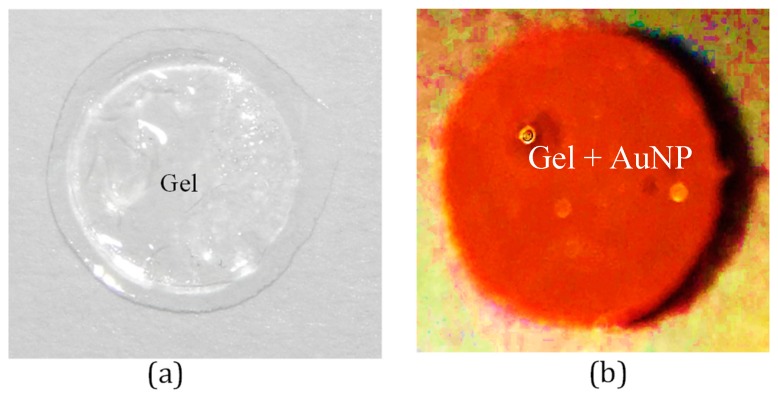
Photograph of the synthesized hydrogel. (**a**) Undoped hydrogel. (**b**) AuNP-doped hydrogel.

The gold nanoparticle-doped (AuNP) hydrogel used in the MOHFET was made by polymerized acrylamide gel as backbones and bis-acrylamide as crosslinks with the gel thickness of the channel being approximately 50 µm. The gold nanoparticle diameter was 50.7 ± 7.1 nm as measured with a transmission electron microscope. Figure 2 shows a picture of the synthesized undoped and AuNP-doped hydrogel.

### 2.2. Impedance Spectroscopy of Hydrogel

**Figure 3:**
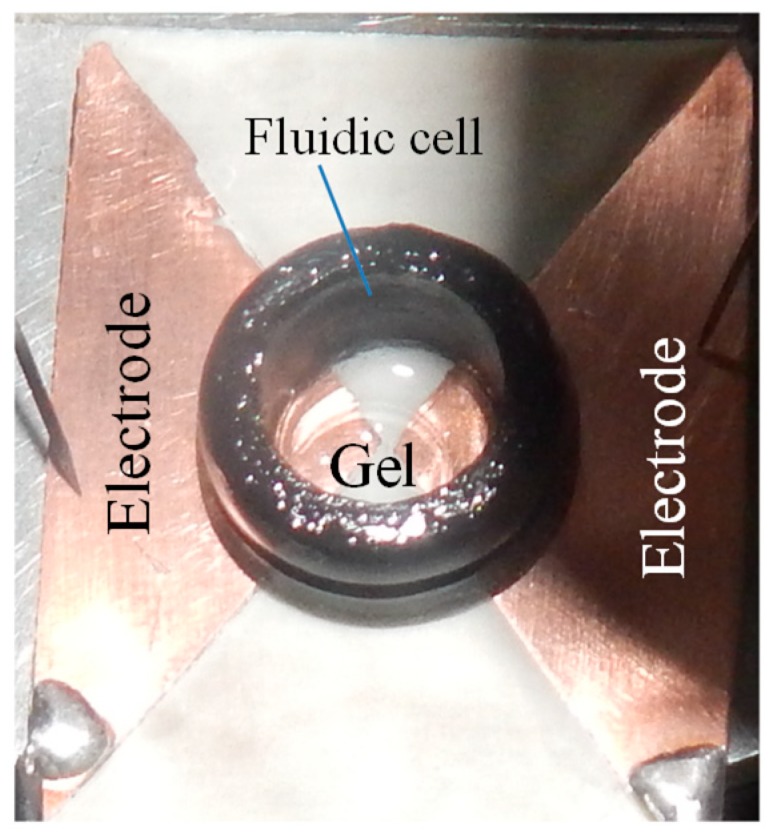
The device used for monitoring impedance changes of the hydrogel as the hydrogel responds to chemical concentrations.

Impedance spectroscopy is a well-established electrochemical technique for measuring hydrogel response to different analytes [8,10,12]. In this paper, impedance spectroscopy was used to measure the impedance change of the synthesized hydrogel as a function of its ionic content. As shown in Figure 3, the hydrogel was placed inside a fluidic cell (volume of 472 μL) centered over the 1 mm wide inter-electrode gap of two planar triangular 35 µm-thick copper electrodes. The substrate for the copper electrodes was a Roger RO4003C hydrocarbon/ceramic material of thickness 1.524 mm from Rogers Corporation (Chandler, AZ, USA). The fluidic cell was glued to the substrate/copper electrodes with epoxy. The sensor terminals were connected to the terminals of a 4284A Hewlett Packard Precision LCR meter (Hewlett Packard, Palo Alto, CA, USA). An analyte was introduced into the sensor cell, and the series capacitance and series resistance of the hydrogel sensor were monitored from 20 Hz to 1 MHz in 25 logarithmic steps.

### 2.3. Contact-Mode Microwave Hydrogel Measurements

**Figure 4:**
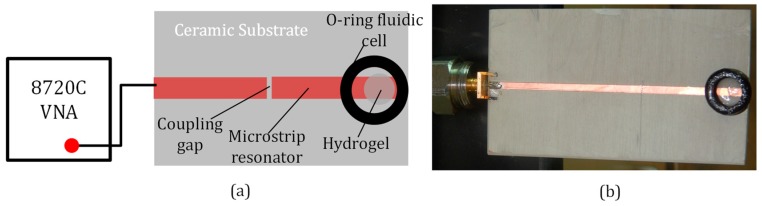
Sensor for monitoring hydrogel response with microwaves in the contact mode. (**a**) Schematic of the sensor. (**b**) Photograph of the sensor.

Resonator-based microwave sensors have been used in the past in material-assisted sensors because resonant microwave circuits are most sensitive at or around their resonance frequencies [19,20,21,22,23]. To demonstrate the use of microwaves for hydrogel response monitoring, a hydrogel-integrated microwave resonator sensor was developed. This sensor consisted of a planar 1.3412 GHz half-wavelength copper microstrip resonator on a Rogers TMM13i grounded ceramic substrate (Rogers Corporation, Chandler, AZ, USA). The TMM13i ceramic was used in the sensor design because it has a low dielectric loss (loss tangent 0.0019) and was not very porous to the analytes that were used in this study. The relative dielectric constant of the ceramic substrate was 13. A fluidic cell was glued to the open circuit end of a half-wavelength microstrip resonator, and the hydrogel was placed inside the fluidic cell, as shown in Figure 4. The half-wavelength microwave resonator of this sensor was 35.5 mm long and 1.8 mm wide. The other end of the sensor resonator was critically coupled [14,15,16,36] to a 58.5 mm-long microstrip transmission line through a 100 μm coupling gap (Figure 4). The TMM13i dielectric substrate of the resonator was 2.54 mm thick and it has an area of 53 mm × 94 mm. A SubMiniature version A (SMA) connector (Amphenol Corp., Wallingford, CT, USA) was used to connect the resonator to an 8720C HP Vector Network Analyzer (VNA) (Hewlett Packard, CA, USA). The power, number of points, and the intermidate frequency (IF) bandwidth of the VNA were set to 0 dBm, 201, and 3.7 kHz, respectively. A LabVIEW program (LabVIEW version 2016, National Instruments, Austin, TX, USA) was used to automatically monitor the change in probe’s S11 due to the hydrogel response to the DI water and 1× PBS.

### 2.4. Contactless (Remote) Microwave Monitoring of Hyrogel Response

**Figure 5:**
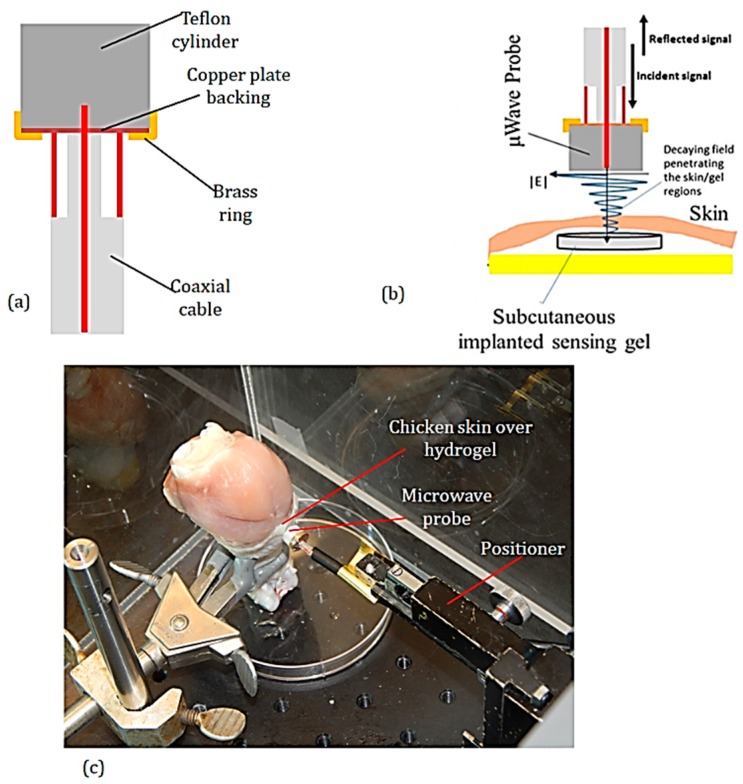
Remote microwave probing of hydrogel. (**a**) Schematic of the Teflon microwave probe for remote microwave monitoring of hydrogel response. (**b**) Schematic of setup for monitoring hydrogel response through chicken skin. The field distribution of the decaying microwave fields is perturbed when hydrogel swells or de-swells. This results in changes in the S_11_ of the probe. (**c**) Photograph of setup for monitoring hydrogel response through the skin of a chicken drumstick.

Microwave coaxial probes have been used in the past to remotely monitor the dielectric properties of materials [37]. The setup for non-contact monitoring of the hydrogel response via remote microwave sensing of hydrogel response is shown in Figure 5. The setup employs a coaxial microwave probe. The probe consists of a Teflon cylinder coupled to a RG 58U coaxial cable of length 50 cm. The Teflon cylinder has a diameter of 8.5 mm and length 5 mm. A brass ring was used to secure a copper back plate to one of the flat surfaces of the Teflon cylinder, as shown in Figure 5a. To transfer the microwave signals from the coaxial cable to the Teflon block, 1 mm length of the center conductor of the coaxial cable was force-fitted into the Teflon cylinder and the outer conductor of the coaxial was soldered to the copper back plate. The coaxial cable of the probe was connected to the 8720C HP Vector Network Analyzer (Hewlett Packard, CA, USA). The power, number of points, and IF bandwidth of the VNA were set to 0 dBm, 201 and 3.7 kHz, respectively.

To remotely monitor the hydrogel response through animal skin, the hydrogel was implanted under the skin of a chicken drumstick. The probe was positioned at a standoff distance of 0.5 mm from the chicken-skin surface over the embedded hydrogel (Figure 5b,c). A syringe and needle were then used to inject water or 1× PBS into the chicken thigh and the microwave probe was used to remotely monitor hydrogel response through the chicken skin. This measurement was done inside a protective Plexiglass box to prevent the chicken thigh sample from drying up. A 0.5 mm standoff distance was chosen in these measurements because 0.5 mm is the resolution of the positioning system used in the test setup. However, the closer the probe is to the container, the stronger the interaction of the microwave with the hydrogel. The manner in which the E-field of the microwaves decay can be seen in the E-field plot in Figure 5b.

### 2.5. Potentiometric Method for Monitoring Hydrogel Response

A potentiometric method for monitoring hydrogel response is a desirable method because it will enable the hydrogel sensor to be integrated with inexpensive voltage readout electronics. Furthermore, it opens up the possibility of using the hydrogel in novel electronic circuits for highly sensitive detection. In the potentiometric method for monitoring hydrogel response, the hydrogel was placed asymmetrically at the inter-electrode gap of the two planar triangular electrodes, as shown in Figure 3, and the electric potential difference across the hydrogel (due to the difference in the ionic concentrations between the two regions where the electrodes make contact with the hydrogel) was measured with a 175A Keithley Multimeter (Keithley Instruments, Cleveland, OH, USA) every 0.02 min over 1× PBS and DI water cycles.

### 2.6. Monitoring Hydrogel Response with MOHFET Current-Voltage Characteristics

**Figure 6:**
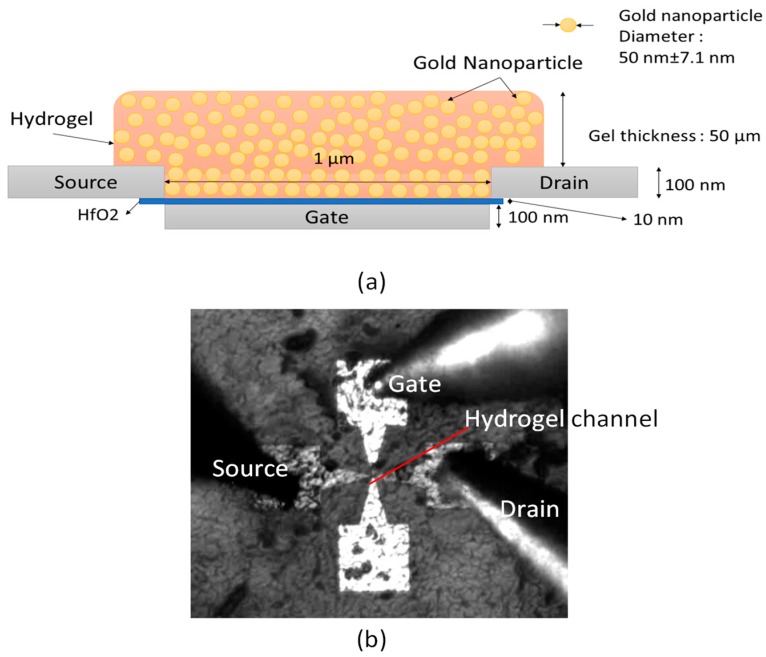
Hydrogel field-effect transistor (FET) sensor. (**a**) Schematic of the sensor. (**b**) Photograph of sensor. The fabricated devices consisted of four electrodes but only three of the electrodes were probed in the actual FET sensor.

Based on the observed potentiometric responses of the hydrogel to analytes from the measurement of the previous section, the hydrogel was used to develop a field-effect transistor (FET) sensor. This hydrogel sensor with FET was designed by using undoped and gold nanoparticle-doped hydrogel as the channel material in an open-channel FET structure. In earlier publications, we have reported the fabrication and characterization of this device, used as a platform for experimenting with different post-deposited channel materials [30,31]. The FET consisted of a bottom-embedded gate with hafnium oxide gate dielectric [30,31] and surface-exposed platinum drain and source electrodes with effective channel length of 1 µm. The drop-cast hydrogel thickness was 50 µm. The schematic and photograph of this FET sensor is shown in Figure 6. The electrodes of the sensor were connected to a 4156A HP Precision Semiconductor Current-Voltage Parameter Analyzer (Hewlett Packard, CA, USA) through a probe station. The experiment with this FET was carried out with various concentrations of PBS (percentage by volume). The IV Characteristics curve of the FET were recorded for gate-source voltage (V_gs_) of −3 V, 0 V and +3 V for drain-source voltage (V_ds_) from −6 Volt to +6 Volt.

## 3. Results

### 3.1. Impedance Spectroscopy for Monitoring Hydrogel Response

**Figure 7:**
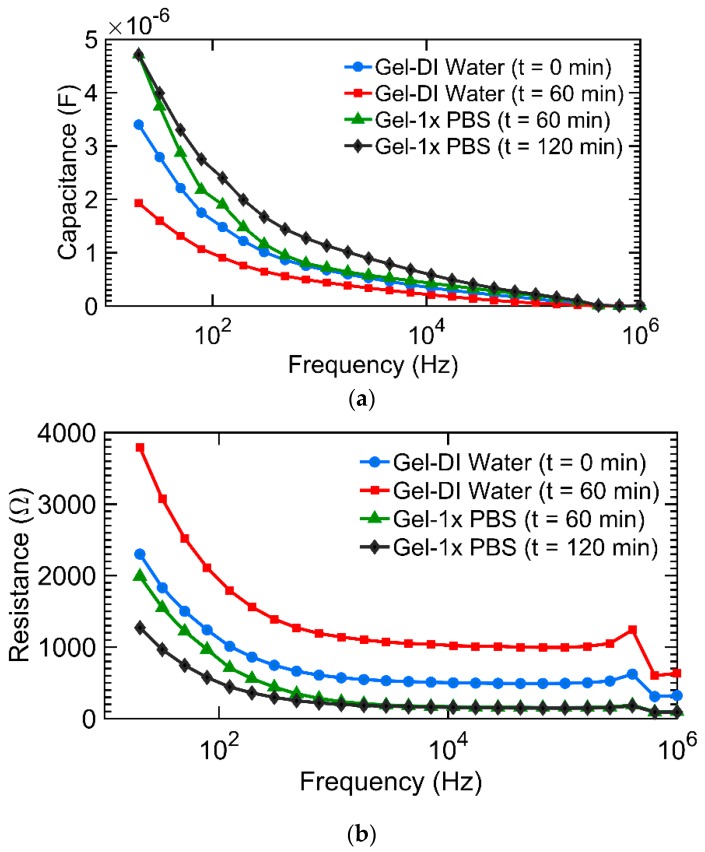
Impedance of the hydrogel-integrated sensor. (**a**) Time-dependent change in capacitance of the hydrogel sensor. (**b**) Time-dependent change in resistance of the hydrogel sensor. It can be seen that phosphate buffered saline (PBS) reduces the hydrogel sensor’s electric resistance and increases hydrogel’s response capacitance.

**Figure 8:**
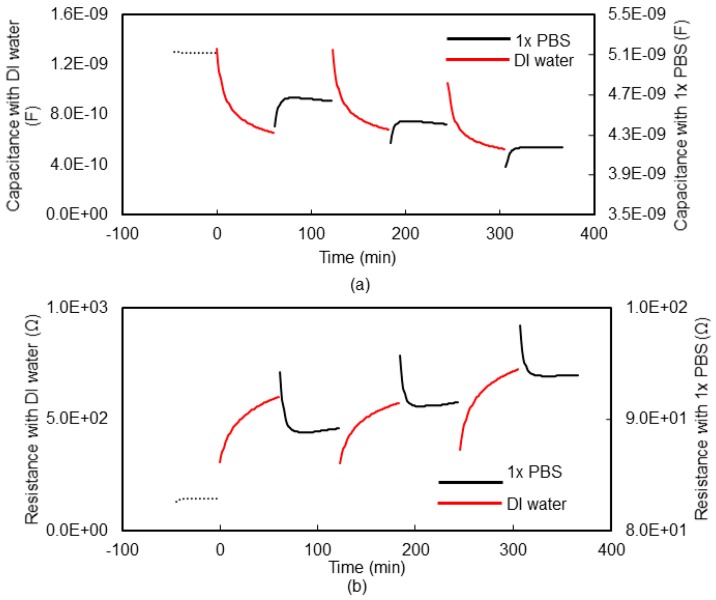
Impedance of the hydrogel sensor as a function of time as solutions with different ionic concentrations were introduced. (**a**) Hydrogel sensor capacitance in response to deionized (DI) water or 1× PBS. (**b**) Hydrogel sensor resistance in response to DI water or 1× PBS.

Figure 7a,b show the measured impedance (capacitance and resistance) of the DI water and 1× PBS of the hydrogel sensor (sensor shown in Figure 3) as a function frequency at different times. The measurements were taken over a frequency range of 20 Hz to 1 MHz at 4 different times. Time t = 0 was when the hydrogel was taken from its original 1× PBS medium, placed at the inter-electrode gap of the fluidic cell, and DI water added to the fluidic cell; time t = 60 min was at 60 min and this was the time when the DI water was siphoned out of the fluidic cell and replaced with 1× PBS and so on, as indicated in the insets of Figure 7. Figure 8a,b shows the result of the continuous monitoring of hydrogel capacitance and resistance over cycles of adding 1× PBS and DI water to the fluidic cell at a single frequency. The single frequency for the continuous monitoring (every 1.28 min) of the hydrogel was 638 kHz. It can be seen from these results that the signature of the hydrogel response to DI water is a decrease in sensor capacitance, while the signature of hydrogel response to 1× PBS is an increase in sensor capacitance. The reverse is the case for sensor resistance.

### 3.2. Contact-Mode Microwave Measurements of Hydrogels

**Figure 9:**
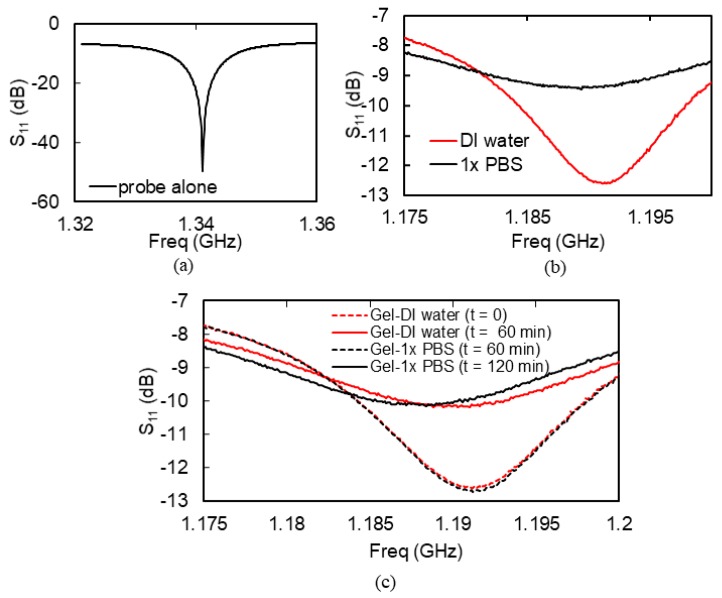
Microwave reflection spectra of the microwave probe. (**a**) Spectrum of the microwave probe with no hydrogel or analyte; (**b**) Spectrum of the microwave probe with analyte but no hydrogel; (**c**) Spectra of the microwave probe with hydrogel for different analytes at different times.

**Figure 10:**
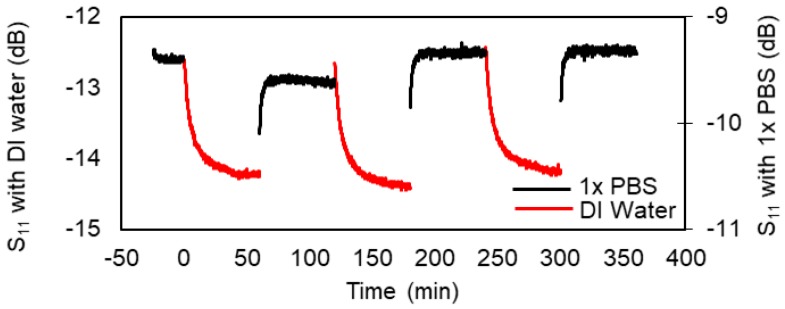
S_11_ of microwave resonator/probe with hydrogel at 1.1917 GHz as a function time with different analytes.

The hydrogel from impedance spectroscopy measurements was used in the contact-microwave hydrogel sensor device shown in Figure 4. The S_11_ spectrum of the microwave resonator probe and fluidic cell assembly before loading the hydrogel and the analyte into the fluidic cell is shown in Figure 9a. Figure 9b shows the S_11_ of the probe assembly when the fluidic cell of the probe was loaded with DI water or with 1× PBS, without hydrogel integration with the resonator. With only the analyte present in the fluidic cell, the S_11_ spectra of the sensor did not change significantly with time. Figure 9c shows the S_11_ of the probe loaded with DI water and 1× PBS, now with the hydrogel at the tip of the resonator inside the fluidic cell. This measurement was done over a frequency band of 1.175 GHz to 1.2 GHz at 4 different times. At time t = 0 the hydrogel was taken from its original 1× PBS medium, placed in the fluidic cell at the tip of the microstrip resonator, and DI water added to the fluidic cell. At time t = 60 min, the DI water was siphoned out of the fluidic cell and replaced with 1× PBS; and so on. Figure 10 shows the result of continuously monitoring (every 6 s) the S_11_ of the hydrogel sensor over cycles of 1× PBS and DI water at a chosen frequency within the 1.175 GHz to 1.2 GHz band. The chosen single frequency for this S_11_ monitoring was 1.1917 GHz. This frequency was chosen because it is the frequency where the S_11_ change of the sensors was maximum when the analyte was changed from DI water to 1× PBS.

It should be noted that the smaller resistance of the PBS + hydrogel loads down the microwave resonator resulting in a shallow S_11_ around the resonance frequency as can be seen in Figure 9b. With the water analyte, however, the hydrogel resistance increases and the S_11_ at resonance becomes much smaller. These observations are corroborated by the impedance measurement results shown in Figure 7.

### 3.3. Remote Microwave Monitoring of Hydrogel Response

**Figure 11:**
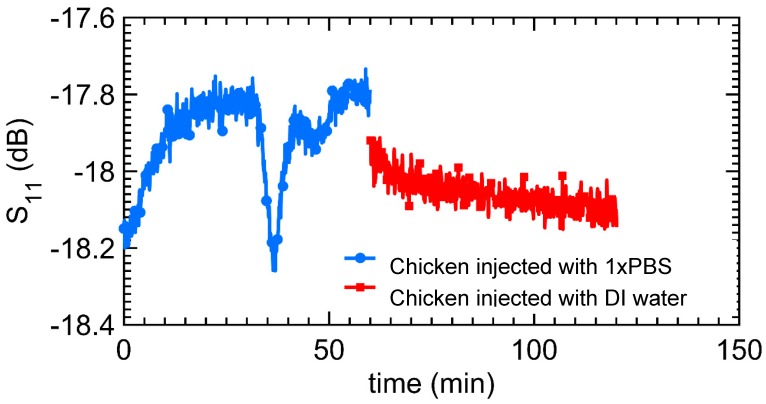
Remote monitoring of the swelling/de-swelling of hydrogel sensor implanted under the skin of a chicken drumstick with the Teflon-protected microwave probe.

Figure 11 shows the result of using the probe to continuously monitor the hydrogel implanted under the skin of a chicken drumstick. The frequency for the remote monitoring was 3.726 GHz. The probe response is more complex than before because of other ionic species inside the chicken. However, the change in the S_11_ is consistent with the results obtained in the contact-mode measurements (Figure 9). In this measurement, at probe resonance, the injection of PBS solution reduces the magnitude of S_11_ while the injection of water increases S_11_ magnitude.

### 3.4. Potentiometric Method for Monitoring Hydrogel Response

**Figure 12:**
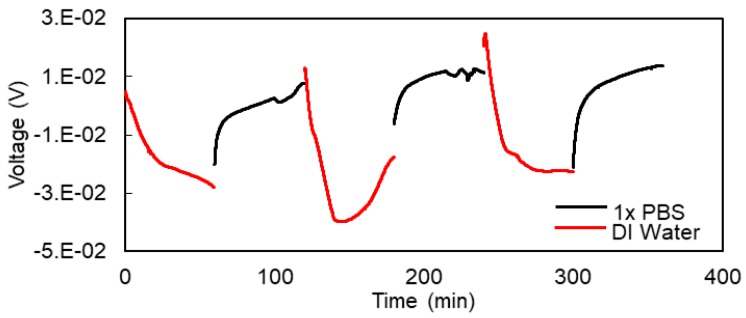
Potential generated across the hydrogel sensor as a function of time in response to 1× PBS and DI water.

The voltage difference across the hydrogel in response to different analytes was measured as a function of time. As shown in Figure 12, the potentiometric response to the hydrogel to DI water and 1× PBS was measured. The interesting aspect of this measurement is that it can be accomplished using a simple digital voltmeter. It does not require any additional signal source or sensitive detection equipment Moreover, it can be used as the basis of a voltage-controlled hydrogel oscillator for wireless telemetry of hydrogel response to chemicals. The normalized change in the voltage (∆V/V) is very large, at around 500%.

### 3.5. Montioring Hydrogel Response with Metal-Oxide-Hydrogel Field-Effect Transistor (MOHFET) Current-Voltage (IV) Characteristics

**Figure 13:**
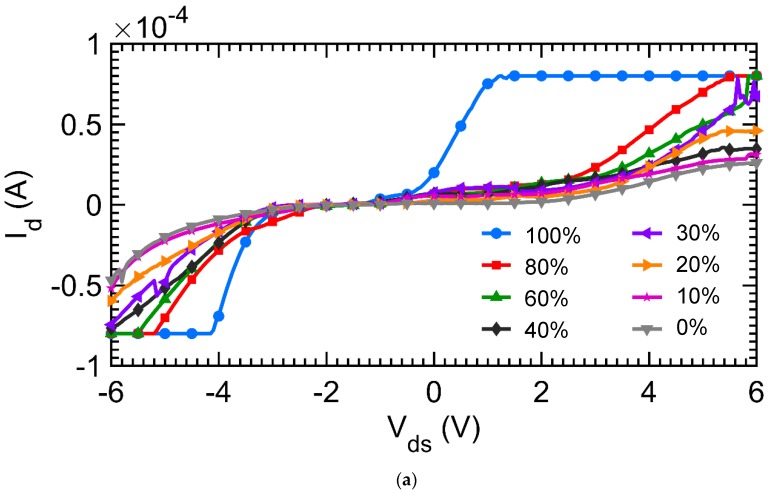

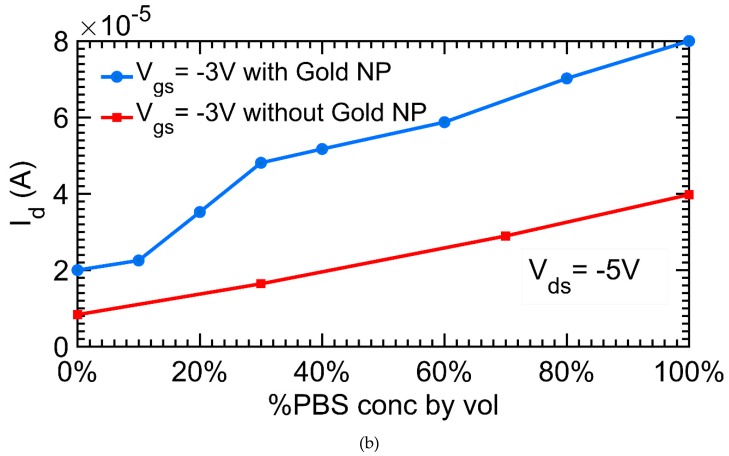
Chemical sensing with the hydrogel FET (**a**) I_d_-V_ds_ plot of the hydrogel with Au-NP device with various concentrations of PBS in DI water by volume at V_gs_ = −3 V and V_ds_ = −6 V to +6 V (**b**) I_d_ at V_ds_ = −5 V for different concentrations of PBS by volume for V_gs_ = −3 V.

The hydrogel FET sensor was characterized with various concentrations of phosphate buffer solution (PBS). The Current-Voltage (IV) characteristics were measured for gate voltage V_gs_ = −3, 0 and +3 V for drain to source voltage V_ds_ from −6 Volt to +6 Volt as shown in Figure 13. The percentage changes in the channel conductance with and without PBS was around 120%, which is similar to the resistance changes discussed in the potentiometric measurements.

## 4. Discussion

### 4.1. Microwave Monitoring of Hydrogel Response

As mentioned earlier, hydrogel responds to changes in ionic concentration by absorbing or releasing water (swelling or de-swelling). In the hydrogel integrated with the contact-mode microwave hydrogel sensors, the absorption/release of water leads to changes in the dielectric constant of the hydrogel, and this change can be computed with the Maxwell–Garnett approximation [38]:

(1) $$\varepsilon_{eff}=\varepsilon_{m}\frac{\left(2\delta_{i}\left(\varepsilon_{i}-\varepsilon_{m}\right)+\varepsilon_{i}+2\varepsilon_{m}\right)}{2\varepsilon_{m}+\varepsilon_{i}+\delta_{i}\left(\varepsilon_{m}-\varepsilon_{i}\right)}$$

where *ε_eff_* is the dielectric constant of the swollen or de-swollen hydrogel, *ε_m_* is the dielectric constant of the hydrogel matrix, *ε_i_* is the dielectric constant of the water contained in the hydrogel, and *δ_i_* is the volume fraction of water contained in the hydrogel. Since the hydrogel is in close proximity with the microwave probe, changes in hydrogel dielectric property result in changes in the probe’s response. In the hydrogel sensor with contact-mode microwave readout, the hydrogel was placed at the tip of the microstrip resonator. This tip is where the electric field of the resonator is maximum and where slight changes in the hydrogel’s complex permittivity result in big changes in the microwave impedance of the probe.

**Figure 14:**
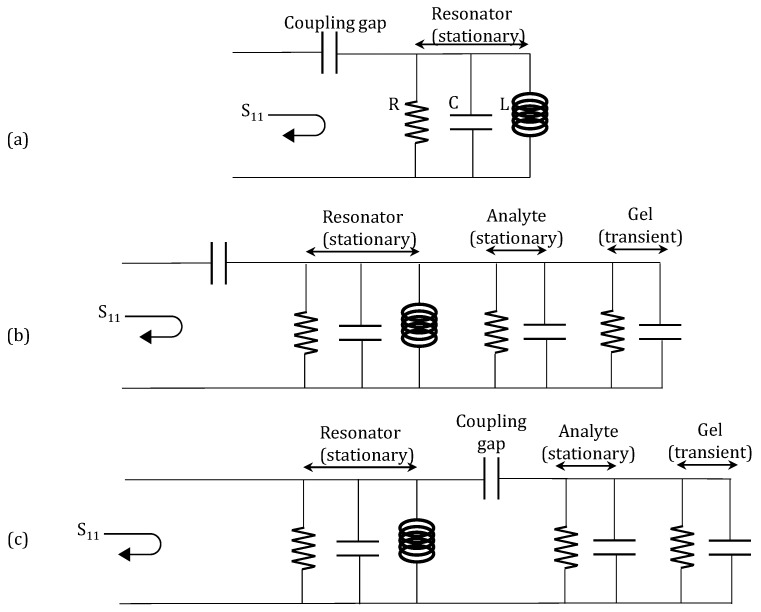
Circuit model of hydrogel sensing with microwave. (**a**) Circuit model of the microstrip microwave resonator; (**b**) Circuit model of the resonator, analyte and hydrogel of the hydrogel sensor with contact-mode microwave readout; (**c**) Circuit model of the probe, analyte and hydrogel of hydrogel sensing with remote microwave readout.

The open-circuit half-wavelength resonator of the hydrogel probe can be modeled as a parallel resonant resistor-capacitor-inductor (RCL) circuit [36] as shown in the model of Figure 14. In this model, the hydrogel and the analyte are modeled as an RC circuit [39]. The microwave resonator and the analyte are modeled with constant circuit parameters because their properties do not change over time in the measurements. However, the hydrogel’s electrical parameters change in a time-dependent manner since the hydrogel respond slowly to changes in the concentration of an analyte in contact with the hydrogel (as the hydrogel gradually swells or de-swells). In addition to the hydrogel, the background analyte also loads the resonator. However, the analyte loads the hydrogel instantaneously and the loading effect is stationary.

The hydrogel response sensing with remote microwave probe can be modeled in a similar way as the contact-mode sensor. However, the dielectric layers (air, plastic, or skin) between the hydrogel and the microwave probe are modeled by a coupling capacitor as shown in Figure 14c. In the subcutaneously implanted hydrogel, due to the low coupling capacitance between the probe and the implanted hydrogel, the observable change in probe S_11_ due to the swelling/de-swelling of the hydrogel is low. This is seen by the small variation in the S_11_ in Figure 11 as the hydrogel swells/de-swells. Furthermore, the environment of the hydrogel when the hydrogel was implanted under the chicken skin differs significantly from the controlled fluidic cell of the hydrogel in the contact-mode microwave measurement. Hence, there is a noticeable artifact in Figure 11 of the S_11_ of the probe in response to the swelling/de-swelling of the hydrogel under the skin of the chicken.

**Figure 15:**
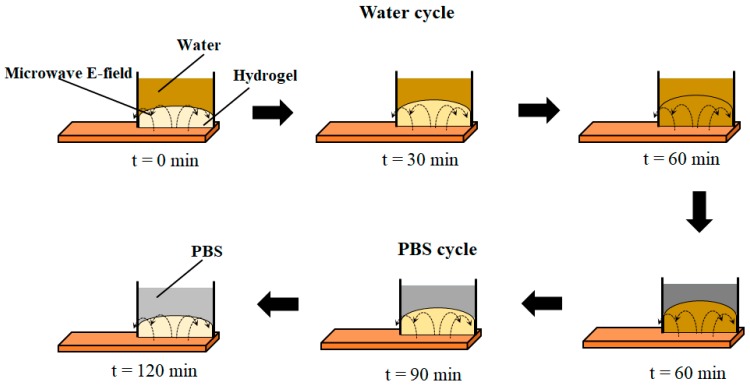
Schematic illustrating the behavior of a hydrogel as an encapsulation medium for water.

From the results presented in Section 3.2 and Section 3.3 and from the analysis above, it can be concluded that the hydrogel responds to the analyte because it is a water encapsulation medium whose encapsulation property is time-dependent. When the hydrogel swells by absorbing water, it brings more water closer to the microwave probe. Therefore, the S_11_ measured by the probe varies in a time-dependent manner towards the S_11_ of the probe when the probe is sensing only water (without the hydrogel present). The reverse occurs when the hydrogel de-swells. The schematic illustrating the hydrogel as a transient encapsulation medium for the hydrogel sensors with contact-mode microwave is shown in Figure 15. The degree to which the fields of the probe interact with the hydrogel/analytes can be seen in the figure. This illustration is easily extended to the remote microwave readout case. This illustration also applies to the results of the hydrogel impedance sensor of Section 3.1.

In future studies, the contact and remote microwave probes will be calibrated to different chemicals, including glucose, in order to compare their sensitivities to that of other transduction techniques for hydrogel sensing. It should be mentioned that the microwave transduction technique has the advantage that its sensitivity can be easily amplified with innovative detection techniques such as the heterodyne detection technique [33]. Developing a highly-sensitive transduction technique for hydrogel sensing will allow researchers to focus more on synthesizing hydrogels for chemical and biomolecule selectivity rather than focusing on synthesizing for both sensitivity and selectivity at the same time.

### 4.2. Montioring Hydrogel Response with MOHFET IV Characteristics

The predominant conduction mechanism in hydrogel film is ionic conduction [40]. A hydrogel doped with Au-NP increases the current conduction limit due to additional conductivity of gold nanoparticles and possible current tunneling between them. The channel conductivity increased as a function of PBS concentration in both hydrogel and AuNP-hydrogel channels but the change was larger in AuNP-hydrogel channel. At zero PBS concentrations the channel conductivities of both devices were low and similar, indicating that the hydrogel was swollen and any additional conductivity due to AuNP was lost. The MOHFET data will be used to design an implantable oscillator that can wirelessly [41] send glucose concentration [42] to an outside reader unit.

## 5. Conclusions

Two novel methods for monitoring the response of hydrogels to chemicals have been developed in this paper. The first method is the microwave-monitoring method. This method measures the perturbation of the fields of a microwave probe by swelling/de-swelling hydrogel to monitor hydrogel response. This allows both contact (invasive) and remote (non-invasive) monitoring of the hydrogel response. The remote microwave sensing is of greater interest because it can be used for sensing the response of subcutaneously implanted hydrogels. The other novel method is the field-effect transistor method in which the current-voltage characteristics of a FET with a hydrogel channel was used to monitor the response of the hydrogel to chemicals. The FET monitoring is an extension of the potentiometric measurement that was also presented in this paper. The sensitivity of the potentiometric technique was 3 times larger than the resistance and MOHFET changes (Figure 7) and 35 times larger than the S_11_ changes (Figure 10). The FET sensing technique has the advantage that it can be used in an oscillator for wireless telemetry of hydrogel response to chemicals.

## References

[1] Guenther M., Gerlach G. Hydrogels for Chemical Sensors. https://link.springer.com/chapter/10.1007/978-3-540-75645-3_5.

[2] Lin G., Chang S., Hao H., Tathireddy P., Orthner M., Magda J., Solzbacher F. (2010). Osmotic Swelling Pressure Response of Smart Hydrogels Suitable for Chronically-Implantable Glucose Sensors. Sens. Actuators B Chem..

[3] Richter A., Paschew G., Klatt S., Lienig J., Arndt K.F., Adler H.P. (2008). Review on Hydrogel-based pH Sensors and microsensors. Sensors.

[4] Cong J., Zhang X., Chen K., Xu J. (2002). Fiber optic Bragg grating sensor based on hydrogels for measuring salinity. Sens. Actuators B Chem..

[5] Kuzimenkova M.V., Ivanov A.E., Thammakhet C., Mikhalovska L.I., Galaev I.Y., Thavarungkul P., Kanatharana P., Mattiasson B. (2008). Optical responses, permeability and diol-specific reactivity of thin polyacrylamide gels containing immobilized phenylboronic acid. Polymer.

[6] Tierney S., Volden S., Stokke B.T. (2009). Glucose sensors based on a responsive gel incorporated as a Fabry-Perot cavity on a fiber-optic readout platform. Biosens. Bioelectron..

[7] Tierney S., Hjelme D.R., Stokke B.T. (2008). Determination of swelling of responsive gels with nanometer resolution. Fiber-optic based platform for hydrogels as signal transducers. Anal. Chem..

[8] Sheppard N.F., Salehi-Had S., Tucker R.C. (1991). Design of a conductimetric microsensor based on pH-sensitive polymer hydrogels. Proc. Annu. Conf. Eng. Med. Biol..

[9] Huang H.M., Liu C.H., Lee V., Lee C., Wang M.J. (2010). Highly sensitive glucose biosensor based on CF4-plasma-modified interdigital transducer array (IDA) microelectrode. Sens. Actuators B Chem..

[10] Guan T., Ceyssens F., Puers R. (2012). Fabrication and testing of a MEMS platform for characterization of stimuli-sensitive hydrogels. J. Micromech. Microeng..

[11] Kikuchi A., Suzuki K., Okabayashi O., Hoshino H., Kataoka K., Sakurai Y., Okano T. (1996). Glucose-sensing electrode coated with polymer complex gel containing phenylboronic acid. Anal. Chem..

[12] Mac Kenna N., Calvert P., Morrin A. (2015). Impedimetric transduction of swelling in pH-responsive hydrogels. Analyst.

[13] Tabib-Azar M., Leclair S.R. (2000). Applications of evanescent microwave probes in gas and chemical sensors. Sens. Actuators B Chem..

[14] Tabib-Azar M., Wang R. (2001). Planar evanescent microwave imaging probes for nondestructive evaluation of materials with very high spatial resolutions and scan rates. AIP Conf. Proc..

[15] Tabib-Azar M. (2001). Microwave microscopy and its applications. AIP Conf. Proc..

[16] Tabib-Azar M. (2001). Evanescent Microwave Microscope: A New Nondestructive Material Evaluation Tool with Very High Resolutions-Part 2. CSNDT J..

[17] Sinha K., Fawole O.C., Tabib-Azar M. Non-invasive monitoring of electrical parameters of Schefflera arboricola leaf. Proceedings of the 2015 IEEE SENSORS.

[18] García-Valenzuela A., Tabib-Azar M. (1999). Evanescent microwave probes and microscopy. Rev. Mex. Fis..

[19] Jones T.R., Member G.S., Zarifi M.H., Member S. (2017). Miniaturized Quarter-Mode Substrate Integrated Cavity Resonators for Humidity Sensing Miniaturized Quarter-Mode Substrate Integrated Cavity Resonators for Humidity Sensing. IEEE Microw. Wireless Compon. Lett..

[20] Bogner A., Steiner C., Walter S., Kita J., Hagen G., Sensors R.M. (2017). Planar Microstrip Ring Resonators for Microwave-Based Gas Sensing: Design Aspects and Initial Transducers for Humidity and Ammonia Sensing. Sensors.

[21] Rydosz A., Maciak E., Wincza K., Gruszczynski S. (2016). Microwave-based sensors with phthalocyanine films for acetone, ethanol and methanol detection. Sens. Actuators B Chem..

[22] Zarifi M.H., Gholidoust A., Abdolrazzaghi M., Shariaty P., Hashisho Z., Daneshmand M. (2018). Sensitivity enhancement in planar microwave active-resonator using metal organic framework for CO_2_ detection. Sens. Actuators B Chem..

[23] Li H., Chen Z., Borodinov N., Luzinov L., Yu G.J., Wang P.S. (2017). Multi-frequency measurement of volatile organic compounds with a radio-frequency interferometer. IEEE Sens. J..

[24] Fawole O., Sinha K., Tabib-Azar M. Monitoring yeast activation with sugar and zero-calorie sweetener using terahertz waves. Proceedings of the 2015 IEEE SENSORS.

[25] Fawole O.C., Tabib-Azar M. (2016). Terahertz Near-Field Imaging of Biological Samples with Horn Antenna-Excited Probes. IEEE Sens. J..

[26] Wu H., Yu G., Pan L., Liu N., McDowell M.T., Bao Z., Cui Y. (2013). Stable Li-ion battery anodes by in-situ polymerization of conducting hydrogel to conformally coat silicon nanoparticles. Nat. Commun..

[27] Shi Z., Zhao W., Li S., Yang G. (2017). Self-Powered Hydrogel Induced by Ion Transport. Nanoscale.

[28] Dolai S., Leu H.Y., Magda J., Tabib-Azar M. (2018). Hydrogel Gold Nanoparticle Switch. IEEE Electron Device Lett..

[29] Dolai S., Leu H.Y., Magda J., Tabib-Azar M. (2018). Metal-Oxide-Hydrogel Field-Effect Sensor. IEEE Electron Device Lett..

[30] Pai P., Chowdhury F.K., Dang-Tran T.V., Tabib-Azar M. TiOx memristors with variable turn-on voltage using field-effect for non-volatile memory. Proceedings of the 2013 IEEE SENSORS.

[31] Mou N.I., Zhang Y., Pai P., Tabib-Azar M. (2017). Steep sub-threshold current slope (∼2 mV/dec) Pt/Cu2S/Pt gated memristor with lon/Ioff> 100. Solid State Electron..

[32] Thoniyot P., Tan M.J., Karim A.A., Young D.J., Loh X.J. (2015). Nanoparticle–Hydrogel Composites: Concept, Design, and Applications of These Promising, Multi-Functional Materials. Adv. Sci..

[33] Matsumoto A., Sato N., Sakata T., Yoshida R., Kataoka K., Miyahara Y. (2009). Chemical-to-electrical-signal transduction synchronized with smart gel volume phase transition. Adv. Mater..

[34] Orthner M.P., Lin G., Avula M., Buetefisch S., Magda J., Rieth L.W., Solzbacher F. (2010). Hydrogel based sensor arrays (2 × 2) with perforated piezoresistive diaphragms for metabolic monitoring (in vitro). Sens. Actuators B Chem..

[35] Flory P.J. (1953). Principles of Polymer Chemistry.

[36] Pozar D.M. (2009). Microwave Engineering.

[37] Meaney P.M., Gregory A.P., Epstein N.R., Paulsen K.D. (2014). Microwave open-ended coaxial dielectric probe: Interpretation of the sensing volume re-visited. BMC Med. Phys..

[38] Garnett J.M. (1906). VII. Colours in metal glasses, in metallic films, and in metallic solutions.—II. Phil. Trans. R. Soc. Lond. A.

[39] Jilani M., Wen W., Cheong L.Y., Zakariya M.A., Rehman M.Z. (2014). Equivalent circuit modeling of the dielectric loaded microwave biosensor. Radioengineering.

[40] Kataoka H., Saito Y., Sakai T., Quartarone E., Mustarelli P. (2000). Conduction Mechanisms of PVDF-Type Gel Polymer Electrolytes of Lithium Prepared by a Phase Inversion Process. J. Phys. Chem. B.

[41] Salsbery G., Tabib-Azar M. Wireless sensor for determining the impedance of human skin. Proceedings of the 2016 IEEE SENSORS.

[42] Salsbery G., Tabib-Azar M. Optical sensor for determining concentration of glucose in water. Proceedings of the 2016 IEEE SENSORS.

